# Evaluation of choriocapillaris perfusion changes in chronic central serous chorioretinopathy following Half-Dose photodynamic therapy

**DOI:** 10.1007/s00417-025-06957-9

**Published:** 2025-10-10

**Authors:** Giacomo Boscia, Pasquale Viggiano, Giulia Corradetti, Alba Chiara Termite, Alfonso Savastano, Alberto Quarta, Alessandro Feo, Ceren Soylu, Rodolfo Mastropasqua, Lisa Toto, Francesco Boscia, SriniVas R. Sadda

**Affiliations:** 1 Department of Translational Biomedicine Neuroscience, University of Bari “Aldo Moro”, Bari, Italy; 2 Doheny Eye Institute, Los Angeles, CA USA; 3 University of Chieti Pescara “Gabriele D’annunzio”, Chieti, Italy; 4 Department of Biomedical Sciences, Humanitas University, Pieve Emanuele-Milan, Italy; 5 Department of Ophthalmology, David Geffen School of Medicine, University of California-Los Angeles, Los Angeles, CA USA; 6 150 N Orange Grove Blvd, 100 Stein Plaza Driveway, Pasadena, Los Angeles, CA 90024 USA

**Keywords:** Central serous chorioretinopathy, Half-dose photodynamic therapy, Choroid, Optical coherence tomography angiography, Choriocapillaris

## Abstract

**Background:**

This study aimed to evaluate changes in choriocapillaris (CC) perfusion in patients with chronic central serous chorioretinopathy (cCSC) following treatment with half-dose photodynamic therapy (PDT), using swept-source optical coherence tomography angiography (SS-OCTA).

**Methods:**

This pilot study included patients diagnosed with cCSC who underwent half-dose PDT using the Visulas 690s PDT Laser System (Carl Zeiss Meditec, Inc., Jena, Germany; 689-nm wavelength, 50J/cm², 83s). Fovea-centered OCTA scans (6 × 6mm) were obtained using the Zeiss Plex Elite 9000 SS-OCT system. OCTA assessments were conducted at baseline prior to treatment and 6 months post-treatment. The primary outcome was the percentage of choriocapillaris flow deficits (FD%) on OCTA. Secondary outcomes included best-corrected visual acuity (BCVA), central macular thickness (CMT), and subfoveal choroidal thickness (SFCT).

**Results:**

The study included 23 eyes from 23 patients with cCSC, with a mean age of 46.3 ± 2.1 years, of which 16 out of 23 were men. Significant improvements were observed in all parameters at 6 months. The FD% decreased significantly from 28.9 ± 2.2% at baseline to 26.4 ± 1.9% after 6 months (*p* = 0.023). BCVA improved from 0.28 ± 0.13 LogMAR to 0.22 ± 0.11 LogMAR (*p* = 0.025). Both CMT and SFCT showed significant reductions (*p* < 0.05). After 6-months, SRF was resolved in 21 out of 23 eyes (91.3%) included in the analysis, while the remaining two eyes showed a reduction of SRF.

**Conclusion:**

Half-dose PDT was effective in improving CC perfusion and other morpho-functional parameters in eyes with cCSC. These findings provide valuable insights into the potential mechanisms through which PDT may influence the progression of cCSC.

## Introduction

Central serous chorioretinopathy (CSC) is a retinal condition marked by prolonged serous detachment of the retina, often involving the macula, which can lead to significant vision impairment [1, 2]. The pathogenesis of CSC is believed to involve abnormalities of both the choroid and retinal pigment epithelium (RPE) [3]. The disease is considered part of the “pachychoroid” disease spectrum, characterized by pathological dilation of the Haller’s layer vessels, leading to choroidal vascular hyperpermeability and accumulation of subretinal fluid (SRF) [1, 4].

The choroidal vascular dysfunction and in particular its impact on the choriocapillaris (CC) has been a topic of recent focus [5, 6]. Several studies indicate that changes in choroidal blood flow, including expanded vessels in Haller’s layer and thinning of the inner choroid, play a key role in the development of CSC [5, 6, 7]. These vascular alterations can result in ischemia of the CC and disruption of the RPE barrier, leading to serous retinal detachment, macular edema, and progressive vision loss [5]. The CC ischemia may be the primary driver of the development of RPE alterations (“pachychoroid pigment epitheliopathy”) and the development of a neovascular response (macular neovascularization, MNV) [8].

Although the exact mechanisms remain uncertain, corticosteroid use, overexpression of the mineralocorticoid receptor (MR) and stress are recognized triggers [9]. Other risk factors include pregnancy and a type “a” personality [10]. The original classification of CSC defined an acute form (aCSC), presenting with SRF accumulation which typically regressed spontaneously within the first 3 months, and a chronic form (cCSC), where the exudation persisted beyond 3 to 4 months, often resulting in irreversible vision damage if untreated [1, 10]. Recently, a new classification system that proposes the definition of the disorder based on its extent (simple:≤2 disc area; complex: >2 disc area) and duration (sporadic: <6 months; persistent: >6 months) has been published [11].

Photodynamic therapy (PDT) is considered the gold standard for CSC treatment. PDT achieves this by targeting the choroidal vasculature, reducing vascular leakage and encouraging healing of the RPE barrier [12, 13]. Advanced imaging technologies such as swept-source optical coherence tomography angiography (SS-OCTA) now allow for non-invasive, high-resolution visualization of both retinal and inner choroidal blood vessels [2, 14, 15]. This technology is particularly effective in evaluating changes in CC perfusion, such as flow deficits, which are considered indicative of disease severity [2].

While PDT is widely accepted as a primary treatment option, the impact of PDT on CC perfusion post-treatment using SS-OCTA remains largely unexplored. Understanding these effects is particularly crucial given the ongoing discourse regarding PDT’s effects on the choroidal vasculature. This study aims to investigate the changes in choriocapillaris perfusion following half-dose PDT (HD-PDT) using SS-OCTA, addressing this important knowledge gap in the context of current controversies surrounding PDT’s impact on the choroid.

## METHODS

### Study participants

This pilot retrospective study included 23 patients diagnosed with cCSC who received treatment with half-dose PDT. The study was conducted at multiple centers, including the Doheny Eye Institute, Pasadena, California, USA; the Department of Ophthalmology of the University of Bari, Bari, Italy; and the Department of Ophthalmology of the University of Chieti, Chieti, Italy, between June 2022 and June 2024. This study is a retrospective observational analysis of patients who were previously treated in routine clinical practice. All data were collected from existing medical records and fully anonymized prior to analysis. In accordance with Italian regulations, no active intervention was performed and no identifiable patient data were used; therefore, approval from an Ethics Committee was not required. However, a written informed consent was obtained from all subjects.

Inclusion criteria for the study were patients with cCSC presenting with persistent SRF lasting for at least 6 months of observation. Exclusion criteria were: (i) patients with any other retinal or choroidal diseases, (ii) patients with an history of previous laser therapy, and (iii) patients who had undergone prior PDT treatment. The patients included in the analysis underwent observation for the first 6 months to evaluate for fluid resolution. After the persistence of fluid for 6 months, they were diagnosed for cCSC and an HD-PDT treatment was planned [11, 13]. All patients underwent a standardized multimodal imaging protocol at baseline, including fluorescein angiography (FA), indocyanine green angiography (ICGA), fundus autofluorescence (FAF), optical coherence tomography (OCT), and OCT angiography (OCTA). At the 6-month follow-up, SS-OCTA and structural OCT were repeated to evaluate treatment outcomes.

### Treatment protocol

ICGA-guided half-dose PDT (3 mg/m^2^) was performed using the Visulas 690 s PDT Laser System (Carl Zeiss Meditec, Inc., Jena, Germany) with a 689-nm wavelength, 50 J/cm² fluence, and a duration of 83 s [16]. The PDT spot was applied to the macular area, specifically targeting the site of choroidal hyperpermeability as identified by ICGA [16].

### Examinations

SS-OCTA imaging was performed using the Zeiss Plex Elite 9000 system (Carl Zeiss Meditec, Inc.). Fovea-centered 6 × 6 mm OCTA scans were acquired at baseline and 6 months post-treatment. FastTrack motion correction software was applied. Images with poor quality due to low signal, incorrect or incomplete segmentation, and/or motion/shadowing artifacts were not included in the analysis. Resultant *en face* OCTA images were used to calculate the CCFD%, which served as the primary outcome measure. The final images were exported for further analysis. Secondary outcomes included best-corrected visual acuity (BCVA), central macular thickness (CMT), and subfoveal choroidal thickness (SFCT), all of which were measured using the structural OCT scans. All the latter parameters were measured at baseline and 6 months after the treatment and were obtained from OCT scans from the Spectralis HRA + OCT (20 × 20 degrees; 512 × 49; fovea-centered; average real-time averaging = 20; Heidelberg Engineering; Heidelberg, Germany). For SFCT analysis the enhanced-depth imaging (EDI) mode was used to better visualize the choroid-sclera interface. BCVA was measured and converted into the LogMar scale. SFCT was measured perpendicularly to Bruch’s membrane (BM), and measured from BM to the choroid-scleral interface at the foveal center [4]. CMT measurements were automatically averaged within the 1-mm-diameter central fovea subfield of the ETDRS (Early Treatment Diabetic Retinopathy Study) grid [4].

### Image analysis

6 × 6 mm CC *en face* OCTA slabs were used to compute CCFD%. For CC computation, the CC slab boundaries were positioned 4–20 μm below Bruch’s membrane, which was delineated by the semiautomated segmentation algorithm provided by the manufacturer. Exported *en face* OCTA images of the CC (PLEX Elite, 1024 × 1024 pixels; central wavelength of 1050 nm; 100’000 A scans per second) were imported in Fiji ImageJ (software version 1.50; National Institute of Health, Bethesda, MD, USA). As described in many prior publications, signal loss artifacts from overlying superficial retinal vessels were mitigated by excluding these regions using a superficial vessel mask [17, 18, 19]. In addition, as there were concerns that SRF and exudative material could impact visualization of deeper layers, signal compensation was applied as previously described [20]. Subsequently, the CC *en face* OCTA images were binarized using the Phansalkar’s local thresholding method (radius of ~ 20 μm) [21]. Moreover, the flow deficits with a diameter < 24 μm were excluded from the analysis, as they are considered to represent a normal intercapillary distance [22]. The final CCFD% was obtained using the function “Analyze Particles” provided by ImageJ and represents the percentage of flow deficits within the 6 × 6 mm CC *en face* OCTA slab.

### Statistical analysis

Statistical analysis was performed using the Statistical Package for the Social Sciences (SPSS Inc., version 20.0, Chicago, IL). Normality of the data was assessed by using the Shapiro-Wilk test. Data were analyzed using paired t-tests to compare baseline and 6-month outcomes. ​The chosen level of statistical significance was set at *p* < 0.05.

## RESULTS

### Demographics

A total of 23 eyes from 23 patients (mean age 46.3 ± 2.1 years, 7 females) were included in the study. The characteristics of the subjects included in the analysis are shown in Table 1. Significant improvements were noted across all outcome measures at the 6-month follow-up. At the 6-month follow-up SRF was resolved in 21 out 23 eyes (91.3%) included in the analysis, while the remaining two eyes showed a reduction of SRF (Table 2). 

**Table 1:** Characteristics of the subjects included in the analysis

Variables	cCSC (*n* = 23)
Age (years)	46.3 ± 2.1
Gender (female, %)	7 (30.43%)
Smoking (%)	8 (34.78%)
Hypertension (%)	10 (43.47%)
Steroid use (%)	0 (0%)

**Table 2:** BCVA, OCT and OCTA analysis of the patients included in the study

	Baseline	6 months	*p*
BCVA (logMAR)	0.28 ± 0.13	0.22 ± 0.11	*p* = 0.025
CMT (µm)	327.2 ± 112.27	247.23 ± 35.76	*p* = 0.002
SFCT (µm)	332.4 ± 78.2	298.2 ± 36.3	*p* = 0.012
CCFD%	28.9 ± 2.2%	26.4 ± 1.9%	*p* = 0.023

Data are presented as mean ± SD. Significance of the pairwise comparison (p). *BCVA* Best Corrected Visual Acuity, *CMT* Central Macular Thickness, *SFCT* Sub-Foveal Choroidal Thickness, *CCFD*% Choriocapillaris Flow Deficit %

#### Choriocapillaris flow

**Fig. 1:**
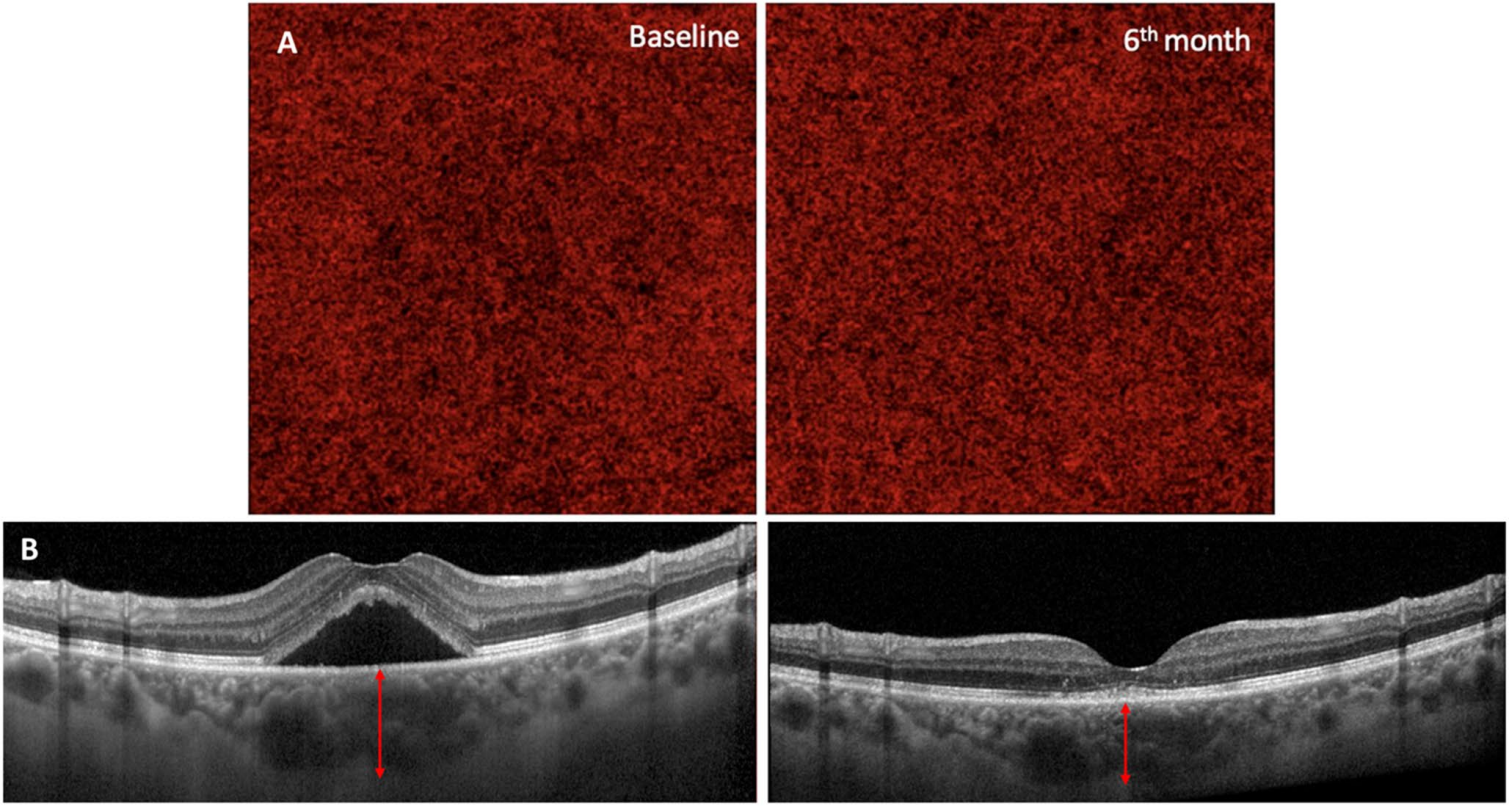
**A** Choriocapillaris (CC) flow deficits in eyes with chronic central serous chorioretinopathy (cCSC) were analyzed using swept-source optical coherence tomography angiography (SS-OCTA) following treatment with half-dose photodynamic therapy (HD-PDT). The CC images were binarized using the Phansalkar method. Scans were performed at baseline and at 6 months. The figure illustrates the changes in CC reperfusion area over the course of the follow-up period. **B** OCT B-scan of the retina and choroid. Sub-foveal choroidal thickness was measured with the caliper function of structural OCT. The SFCT was measured from Bruch’s membrane to the chorio-scleral interface perpendicularly in the center of the fovea (red arrow). Acquisitions were performed at baseline and at 6 months. The figure illustrates the changes in SFCT over the course of the follow-up period

The CCFD% decreased from 28.9 ± 2.2% at baseline to 26.4 ± 1.9% (*p* = 0.023), indicating a reduction in CC flow deficits following half-dose PDT (Fig. 1).

#### BCVA improvements

BCVA improved from 0.28 ± 0.13 LogMAR to 0.22 ± 0.11 LogMAR (*p* = 0.025), reflecting enhanced visual acuity.

#### OCT analysis

CMT showed a significant reduction from 327.2 ± 112.27 μm to 247.23 ± 35.76 μm (*p* = 0.002), highlighting the resolution of the exudation. SFCT analysis revealed a significant reduction from baseline (332.4 ± 78.2 μm) to the 6-month follow-up visit (298.2 ± 36.3 μm, *p* = 0.012) (Fig. 1).

## Discussion

This pilot study aimed to investigate the effects of HD-PDT on CC perfusion, visual acuity, and retinal morphology in patients with cCSC. Using SS-OCTA, we observed significant improvements in multiple outcome measures at the 6-month follow-up. Our findings suggest that HD-PDT may be an effective treatment for reducing choriocapillaris flow deficits, improving BCVA, and resolving fluid accumulation in the retina, subretinal space, and choroid.

The use of SS-OCTA allowed for a detailed, non-invasive assessment of the CC, confirming that PDT helps restore choroidal perfusion by reducing areas of flow deficit [14]. At the 6-month follow-up, SRF was resolved in 21 out 23 eyes (91.3%) included in the analysis. Our findings are consistent with previous studies evaluating choriocapillaris changes after half-dose PDT [23, 24]. Yang et al. and Le et al. used OCTA to demonstrate a reduction in CC flow deficits post-PDT, suggestive of vascular remodeling [25, 26]. Fujita et al. similarly reported CC perfusion improvements [27]. These studies support the hypothesis that HD-PDT can improve CC perfusion by alleviating choroidal congestion.

CC dysfunction in CSC has been previously discussed. Yun and coworkers showed the association between the location of the flow void area and the underlying choroidal vessels thickening [5]. Viggiano and coworkers described a significant reperfusion of the CC in the unaffected fellow eyes of cCSC patients during continuous eplerenone therapy, accompanied by a significant SFCT reduction [7]. The authors speculated that eplerenone had an effect on choroidal vasculature remodeling, leading to a CC mechanical decompression and a subsequent flow restoration [7]. We should note that eplerenone has not been shown to be an effective treatment in larger randomized clinical trials [28]. In the present study, we observed a significant SFCT reduction after HD-PDT treatment (*p* = 0.012). With regards to RPE damage, although FAF was performed at baseline to assess RPE alteration and disease chronicity, it was not used as an outcome measure in this study. The primary objective was to evaluate CC perfusion changes using SS-OCTA.

PDT has been widely used in the management of CSC, with the primary goal of reducing choroidal hyperpermeability and congestion, which are thought to contribute significantly to the disease’s pathophysiology [13, 29]. PDT uses a photosensitive agent, usually verteporfin, which is activated by laser light to target and transiently occlude the choroidal vasculature. This process leads to reduced choroidal thickness and improved retinal fluid dynamics [29]. HD-PDT minimizes the risk of excessive ischemia and occlusion, offering a safer option while still effectively reducing choroidal congestion [30, 31]. The observed reduction in SFCT and CCFD% in our study supports this notion.

The reduction in SFCT suggests that HD-PDT may alleviate choroidal congestion, which is thought to play a critical role in the pathogenesis of CSC. Our result aligns with previous studies demonstrating the effects of HD-PDT on the choroidal vasculature [23, 32, 33]. Nishigori and colleagues described the effects of reduced-fluence (rf)-PDT on choroidal thickness [33]. They speculated that the lower irradiation intensity of rf-PDT reduces vascular occlusion in the CC, leading to choroidal vascular remodeling without damaging the blood flow within the CC [33].

Similarly, in the present study, we observed a reduction in SFCT and a decrease in CCFD%. Based on these findings, we speculate that HD-PDT may reduce choroidal vessel congestion, which in turn decreases the compression on the CC, potentially reducing the CCFD%. It’s crucial also to highlight that HD-PDT has shown potential benefits over full-dose PDT, particularly in minimizing the risk of CC occlusion and atrophy [13]. By reducing the drug’s overall exposure and light activation, HD-PDT may decrease the likelihood of causing excessive damage to the CC. This approach could result in fewer adverse effects while maintaining therapeutic efficacy [13]. Studies directly comparing HD-PDT with full dose PDT and half-fluence PDT may provide further insight into the impact of these parameters on CC perfusion.

Summarizing our results, HD-PDT appears to provide a promising alternative for managing cCSC, offering a balance between efficacy and safety by improving choroidal circulation while avoiding excessive damage to the choroidal vessels. Further studies are needed to confirm these findings and to better understand the long-term effects of half-dose treatment on choroidal health in CSC patients.

One limitation of our study is its retrospective design, which is subject to inherent biases such as selection and recall biases. Additionally, the relatively small sample size and short follow-up period limit the generalizability of our findings. Further prospective studies with larger cohorts and longer follow-up periods are needed to confirm the long-term efficacy and safety of HD-PDT for treating cCSC. Another limitation is the absence of a control group and the lack of interval visits between baseline and the 6-month follow-up.

In conclusion, our study demonstrates that HD-PDT is a promising treatment for patients with cCSC, with significant improvements in choriocapillaris perfusion, visual acuity, and retinal morphology. The use of SS-OCTA allowed for detailed assessment of the choroidal vasculature, providing insight into the physiologic impact of HD-PDT on the CC. These findings contribute to the growing body of evidence supporting the use of PDT in cCSC and highlight the potential of HD-PDT as an effective and potentially safer alternative to standard-dose treatment. Further research is warranted to confirm these results and explore the long-term benefits of HD-PDT in pCSC management.

## References

[1] Rijssen TJ, Van DEHC, Van, Yzer S et al (2019) Progress in retinal and eye research central serous chorioretinopathy: towards an evidence-based treatment guideline. Prog Retin Eye Res 73:100770 31319157 10.1016/j.preteyeres.2019.07.003

[2] Viggiano P, Boscia G, Sadeghi E et al (2025) Pachychoroid disease spectrum: how multimodal imaging and OCT angiography have improved our knowledge. Prog Retin Eye Res. 10.1016/J.PRETEYERES.2025.101372 40414595 10.1016/j.preteyeres.2025.101372

[3] Cheung CMG, Dansingani KK, Koizumi H et al (2024) Pachychoroid disease: review and update. Eye. 10.1038/s41433-024-03253-439719503

[4] Viggiano P, Boscia G, Borrelli E et al (2024) Micropulse laser versus eplerenone for chronic central serous chorioretinopathy: a 12-month comparison. Ophthalmol Ther 13:3175–3188. 10.1007/s40123-024-01059-x 39460897 10.1007/s40123-024-01059-x PMC11564717

[5] Yun C, Huh J, Ahn SM et al (2019) Choriocapillaris flow features and choroidal vasculature in the fellow eyes of patients with acute central serous chorioretinopathy. Graefes Arch Clin Exp Ophthalmol 257:57–70. 10.1007/s00417-018-4179-2 30397792 10.1007/s00417-018-4179-2

[6] Boscia G, Viggiano P, Marzulli F et al (2023) Continuous eplerenone treatment in chronic central serous chorioretinopathy: long-term results from a pilot study. Clin Ophthalmol 17:2003–2012. 10.2147/OPTH.S411094 37483844 10.2147/OPTH.S411094 PMC10361091

[7] Viggiano P, Boscia G, Borrelli E et al (2023) Choriocapillaris reperfusion in resolved chronic central serous chorioretinopathy treated with eplerenone: long-term effects on the fellow eye. Ophthalmol Ther 12:3199–3210. 10.1007/s40123-023-00816-8 37747638 10.1007/s40123-023-00816-8 PMC10640459

[8] Baek J, Kook L, Lee WK (2019) Choriocapillaris flow impairments in association with pachyvessel in early stages of pachychoroid. Sci Rep 9:5565. 10.1038/s41598-019-42052-w 30944393 10.1038/s41598-019-42052-w PMC6447632

[9] Toto L, Ruggeri ML, Evangelista F et al (2022) Choroidal modifications assessed by means of choroidal vascularity index after oral eplerenone treatment in chronic central serous chorioretinopathy. Eye (Lond). 10.1038/s41433-022-02091-6 35590104 10.1038/s41433-022-02091-6 PMC10101961

[10] Kaye R, Chandra S, Sheth J et al (2020) Central serous chorioretinopathy: an update on risk factors, pathophysiology and imaging modalities. Prog Retin Eye Res 79:100865. 10.1016/j.preteyeres.2020.100865 32407978 10.1016/j.preteyeres.2020.100865

[11] Chhablani J, Cohen FB (2020) Multimodal Imaging-Based central serous chorioretinopathy classi Fi cation. Ophthalmol Retina. 10.1016/j.oret.2020.07.026 33131671 10.1016/j.oret.2020.07.026

[12] Toto L, Ares I, Quarta A et al (2024) Visual and anatomical evaluation of navigated subthreshold micropulse laser versus photodynamic therapy in managing chronic central serous chorioretinopathy. Graefes Arch Clin Exp Ophthalmol. 10.1007/s00417-024-06666-9 39425791 10.1007/s00417-024-06666-9

[13] Feenstra HMA, van Dijk EHC, Cheung CMG et al (2024) Central serous chorioretinopathy: an evidence-based treatment guideline. Prog Retin Eye Res 101:101236. 10.1016/j.preteyeres.2024.101236 38301969 10.1016/j.preteyeres.2024.101236

[14] Nassisi M, Lavia C, Alovisi C et al (2017) Short-term choriocapillaris changes in patients with central serous chorioretinopathy after half-dose photodynamic therapy. Int J Mol Sci. 10.3390/ijms18112468 29156649 10.3390/ijms18112468 PMC5713434

[15] Quaranta-El Maftouhi M, El Maftouhi A, Eandi CM (2015) Chronic central serous chorioretinopathy imaged by optical coherence tomographic angiography. Am J Ophthalmol 160:581–587e1. 10.1016/j.ajo.2015.06.016 26133250 10.1016/j.ajo.2015.06.016

[16] Lai TYY, Chan W-M, Li H et al (2006) Safety enhanced photodynamic therapy with half dose verteporfin for chronic central serous chorioretinopathy: a short term pilot study. Br J Ophthalmol 90:869–874. 10.1136/bjo.2006.090282 16597666 10.1136/bjo.2006.090282 PMC1857171

[17] Viggiano P, Miere A, Borrelli E et al (2023) The impact of diabetic retinopathy on the choriocapillaris in neovascular AMD. Invest Ophthalmol Vis Sci. 10.1167/iovs.64.14.32 37988106 10.1167/iovs.64.14.32 PMC10668630

[18] Boscia G, Bacherini D, Vujosevic S et al (2024) Long-term impact of diabetic retinopathy on response to anti-VEGF treatment in neovascular AMD. Invest Ophthalmol Vis Sci. 10.1167/iovs.65.10.6 39093297 10.1167/iovs.65.10.6 PMC11305436

[19] Boscia G, Gnanaraj R, Corradetti G et al (2025) Assessment of inter-device agreement in quantifying choriocapillaris flow deficits in healthy eyes. Curr Eye Res. 10.1080/02713683.2025.2506524 40405602 10.1080/02713683.2025.2506524

[20] Zhang Q, Zheng F, Motulsky EH et al (2018) A novel strategy for quantifying choriocapillaris flow voids using swept-source OCT angiography. Invest Ophthalmol Vis Sci 59:203–211. 10.1167/iovs.17-22953 29340648 10.1167/iovs.17-22953 PMC5770182

[21] Chu Z, Zhang Q, Gregori G et al (2021) Guidelines for imaging the choriocapillaris using OCT angiography. Am J Ophthalmol 222:92–101. 10.1016/j.ajo.2020.08.045 32891694 10.1016/j.ajo.2020.08.045 PMC7930158

[22] Zhang Q, Shi Y, Zhou H et al (2018) Accurate estimation of choriocapillaris flow deficits beyond normal intercapillary spacing with swept source OCT angiography. Quant Imaging Med Surg 8:658–666. 10.21037/qims.2018.08.10 30211033 10.21037/qims.2018.08.10 PMC6127524

[23] Chan W-M, Lai TYY, Lai RYK et al (2008) Safety enhanced photodynamic therapy for chronic central serous chorioretinopathy: one-year results of a prospective study. Retina 28:85–93. 10.1097/IAE.0b013e318156777f 18185143 10.1097/IAE.0b013e318156777f

[24] Nicoló M, Eandi CM, Alovisi C et al (2014) Half-fluence versus half-dose photodynamic therapy in chronic central serous chorioretinopathy. Am J Ophthalmol 157:1033–1037. 10.1016/j.ajo.2014.01.022 24487046 10.1016/j.ajo.2014.01.022

[25] Yang HS, Kang TG, Park H et al (2020) Quantitative evaluation of choriocapillaris using optical coherence tomography and optical coherence tomography angiography in patients with central serous chorioretinopathy after half-dose photodynamic therapy. PLoS One. 10.1371/JOURNAL.PONE.0227718 31929582 10.1371/journal.pone.0227718 PMC6957295

[26] Le HM, Mrejen S, Sibilia L, Cohen SY (2022) Optical coherence tomography angiography quantification of choriocapillaris blood-flow after half-fluence photodynamic therapy for chronic central serous chorioretinopathy. Graefes Arch Clin Exp Ophthalmol 260:2483–2490. 10.1007/S00417-022-05637-2 35348843 10.1007/s00417-022-05637-2

[27] Fujita K, Kawamura A, Yuzawa M (2017) Choriocapillaris changes imaged by OCT angiography after half-dose photodynamic therapy for chronic central serous chorioretinopathy. Ophthalmic Surg Lasers Imaging Retina 48:302–310. 10.3928/23258160-20170329-04 28419395 10.3928/23258160-20170329-04

[28] Lotery A, Sivaprasad S, O’Connell A et al (2020) Eplerenone for chronic central serous chorioretinopathy in patients with active, previously untreated disease for more than 4 months (VICI): a randomised, double-blind, placebo-controlled trial. Lancet 395:294–303. 10.1016/S0140-6736(19)32981-2 31982075 10.1016/S0140-6736(19)32981-2

[29] Khandhadia S, Thulasidharan S, Hoang NTV et al (2023) Real world outcomes of photodynamic therapy for chronic central serous chorioretinopathy. Eye 37:2548–2553. 10.1038/s41433-022-02370-2 36572748 10.1038/s41433-022-02370-2 PMC10397181

[30] Park W, Kim M, Kim RY, Park Y-H (2019) Comparing effects of photodynamic therapy in central serous chorioretinopathy: full-dose versus half-dose versus half-dose-half-fluence. Graefes Arch Clin Exp Ophthalmol 257:2155–2161. 10.1007/s00417-019-04426-8 31367848 10.1007/s00417-019-04426-8

[31] Zaman M, Mihalache A, Huang RS et al (2025) Safety and efficacy of half-dose and half-fluence photodynamic therapy in chronic central serous chorioretinopathy: a systematic review and meta-analysis. Am J Ophthalmol 271:233–242. 10.1016/j.ajo.2024.11.014 39603314 10.1016/j.ajo.2024.11.014

[32] Sawaguchi S, Terao N, Imanaga N et al (2024) One-year choroidal thickness changes after photodynamic therapy for central serous chorioretinopathy evaluated by widefield optical coherence tomography. Graefes Arch Clin Exp Ophthalmol 262:3805–3814. 10.1007/s00417-024-06578-8 38995353 10.1007/s00417-024-06578-8

[33] Nishigori N, Muraoka Y, Ishikura M et al (2023) Extensive reduction in choroidal thickness after photodynamic therapy in eyes with central serous chorioretinopathy. Sci Rep 13:1–9. 10.1038/s41598-023-37802-w 37407690 10.1038/s41598-023-37802-w PMC10322984

